# Questioning the dichotomy between vegetative state and minimally conscious state: a review of the statistical evidence

**DOI:** 10.3389/fnhum.2014.00865

**Published:** 2014-11-03

**Authors:** Giulia Liberati, Thomas Hünefeldt, Marta Olivetti Belardinelli

**Affiliations:** ^1^Institute of Neuroscience, Université Catholique de LouvainBrussels, Belgium; ^2^ECONA – Interuniversity Centre for Research on Cognitive Processing in Natural and Artificial Systems, “Sapienza” University of RomeRome, Italy; ^3^Department of Philosophy, Catholic University of Eichstätt-IngolstadtEichstätt, Germany; ^4^Department of Psychology, Sapienza, University of RomeRome, Italy

**Keywords:** minimally conscious state, vegetative state, unresponsive wakefulness syndrome, consciousness, brain injury

## Abstract

Given the enormous consequences that the diagnosis of vegetative state (VS) vs. minimally conscious state (MCS) may have for the treatment of patients with disorders of consciousness, it is particularly important to empirically legitimate the distinction between these two discrete levels of consciousness. Therefore, the aim of this contribution is to review all the articles reporting statistical evidence concerning the performance of patients in VS vs. patients in MCS, on behavioral or neurophysiological measures. Twenty-three articles matched these inclusion criteria, and comprised behavioral, electroencephalographic (EEG), positron emission tomography (PET) and magnetic resonance imaging (MRI) measures. The analysis of these articles yielded 47 different statistical findings. More than half of these findings (*n* = 24) did not reveal any statistically significant difference between VS and MCS. Overall, there was no combination of variables that allowed reliably discriminating between VS and MCS. This pattern of results casts doubt on the empirical validity of the distinction between VS and MCS.

## Introduction

The vegetative state (VS; Jennett and Plum, 1972), in the past called apallic syndrome (Kretschmer, 1940; Gerstenbrand, 1977), and more recently defined as unresponsive wakefulness syndrome (UWS; Laureys et al., 2010), is a clinical condition that often follows coma, in which the patient appears to be wakeful, with cycles of eye closure and opening resembling those of sleep and waking, but with no signs of awareness, defined in terms of “purposeful or voluntary responses” (Working Group of Royal College of Physicians, 1996; Giacino, 1997; Jennett, 2002). As the diagnosis of VS is entirely based on negative evidence, namely the lack of “purposeful and voluntary responses” (Wade and Johnston, 1999; Kotchoubey et al., 2005), it leads to a high rate of errors (Childs et al., 1993; Andrews et al., 1996; Wijdicks, 2006).

Some severely brain-damaged patients occasionally present weak and inconsistent “purposeful or voluntary responses”. These patients have been defined as in minimally conscious state (MCS), and their limited but definite behaviors are considered evidence of self or environmental awareness (American Congress for Rehabilitation Medicine, 1995; Giacino et al., 2002). Hence, the clinical differentiation between VS and MCS is based on the subtle distinction, based on the evaluator’s interpretation, between purposeless and involuntary behaviors in VS, and purposeful and voluntary behaviors in MCS (Kotchoubey et al., 2005).

In 2002, as a consequence of the lack of data to establish evidence-based guidelines for the diagnosis, prognosis, and management of the MCS, the Aspen Workgroup (Giacino et al., 2002) formulated a consensus-based case definition using behaviorally referenced diagnostic criteria. According to these criteria, MCS should be clearly discernible from VS on the basis of specific behavioral features, such as the ability to follow simple commands, gestural or verbal responses, and purposeful behavior not due to reflexive activity (e.g., responses to questions, reaching for objects, touching or holding objects while accommodating their size and shape, eye movements or sustained fixation in direct response to salient stimuli, etc.).

Several diagnostic tools are commonly used for the diagnosis of patients with disorders of consciousness (Schnakers, 2012). The Glasgow Coma Scale (GCS; Teasdale and Jennett, 1974) was the first validated scale for the assessment of level of consciousness, and measures arousal level, motor function, and verbal abilities (Schnakers, 2012). The Disability Rating Scale (DRS; Rappaport et al., 1982) was developed to rate the effects of traumatic brain injury, to track the patient’s rehabilitation progress, and to estimate how long recovery might take. The JFK Coma Recovery Scale (CRS; Giacino et al., 1991), also in its revised form (CRS-R; Giacino et al., 2004), is one of the most frequently used scales, which addresses auditory, visual, oromotor, communication and arousal functions, arranged hierarchically from simple reflexive activity to complex cognitively-mediated behaviors. Other assessment scales include the Full Outline of UnResponsiveness scale (FOUR; Wijdicks et al., 2005), the Wessex Head Injury Matrix (WHIM; Shiel et al., 2000), and the Sensory Modality Assessment and Rehabilitation Technique (SMART; Gill-Thwaites, 1997).

Albeit the variety of diagnostic tools, the discrimination between VS and MCS remains quite challenging, and the line of demarcation between the two conditions is still not clear-cut. In fact, the misdiagnosis between the two populations appears to be common, and has recently received considerable attention from the media (Hopkin, 2006; Wijdicks, 2006), given its enormous implications in prognosis and treatment. As observed by Gill-Thwaites (2006), several factors render the differential diagnosis of VS and MCS difficult, including the expertise of the evaluators, and the variability in the patients’ medical and physical management. Moreover, the movements of brain-damaged patients can be very limited, inconsistent, and easily exhausted (Gosseries et al., 2011), and are strongly influenced by the fluctuations of the patients’ conditions (Majerus et al., 2005; Schnakers et al., 2009).

With the present review article, we question the empirical validity of the distinction between VS and MCS. In order to shed light on whether this dichotomy is legitimate, we analyzed research articles reporting statistical evidence of the different behavioral and neurophysiological performances of patients with a VS and a MCS diagnosis.

## Methods

To review all studies directly comparing behavioral and neurophysiological responses in MCS and VS patients, we used the **Keywords**: “VS” and “MCS” on PubMed, limiting the search to title and/or abstract. In addition, given the multiple denominations of the VS, a similar search was also performed using the **Keywords**: “UWS” and “MCS” (leading to only one additional reference), and the keywords “apallic syndrome” and “MCS” (with no additional results). These initial searches led to 262 results. We afterwards selected the articles based on our aims. To be included in the review, the articles had to be published on peer-reviewed journals in English, and had to present a direct statistical comparison of behavioral and/or neurophysiological responses in patients in MCS and VS. This led us to discard a large number of studies involving both groups of patients, which however did not report a direct statistical comparison between the two groups, e.g., due to a limited sample of subjects. A total of 23 research articles were finally included in the review, for a total of 47 distinct experiments. Each experiment was analyzed in terms of the type of experimental assessment and the type of stimulation. We divided the different studies in four macro-categories: (1) Stimulus-independent physiological and neurophysiological assessments; (2) Stimulus-dependent behavioral assessments; (3) Stimulus-dependent neurophysiological assessments (using sensory stimuli); and (4) Stimulus-dependent neurophysiological assessments (using meaningful stimuli). These macro-categories, further divided according to the type of stimulation used, are described in the following paragraphs.

Tables s 1–4 summarize the different assessments, diagnostic tools, numbers of subjects, type of measurement, and main outcomes concerning MCS/VS differentiation, for each macro-category.

**Table 1:** **Stimulus-independent physiological and neurophysiological assessments**.

Study	Number of subjects		Diagnostic criteria	Type of assessment	Difference between VS and MCS
	VS	MCS			
Bonfiglio et al. (2013)	4	5	CRS-R	EEG resting state	n.s.
Bonfiglio et al. (2014)	4	5	CRS-R	EEG resting state	Lower alpha (*p* < 0.01) and beta (*p* < 0.05) band power in VS compared to MCS
Coleman et al. (2005)	6	4	RCP; Giacino et al. (2002)	PET resting state	n.s.
Coleman et al. (2005)	6	4	RCP; Giacino et al. (2002)	EEG resting state	Higher degree of slow wave activity in VS (*p* < 0.05)
Cruse et al. (2013)	18	37	CRS-R	Actigraphy sleep assessment	Weaker circadian rhythms in VS (*p* < 0.01)
de Biase et al. (2014)	27	5	CRS-R	EEG sleep assessment	Simultaneous presence of sleep elements less frequent in VS (*p* < 0.001); REM sleep less frequent in VS (*p* < 0.001)
Fernández-Espejo et al. (2011)	10	15	M-STFR (1994); Giacino et al. (2002)	DTI resting state	Higher decrease in mean diffusivity in VS (*p* < 0.006)
Lechinger et al. (2013)	8	9	CRS-R	EEG resting state	n.s.
Lehembre et al. (2012)	10	18	CRS-R	EEG resting state	Higher power in delta band and lower power in alpha band in VS (*p* < 0.05); higher connectivity in MCS (*p* < 0.05)
Schnakers et al. (2008)	13	30	CRS-R, GCS	EEG resting state	Lower BIS in VS compared to MCS (*p* < 0.001)
Wu et al. (2011b)	21	16	M-STFR (1994); Giacino et al. (2002)	EEG resting state	n.s.
Wu et al. (2011a)	20	30	M-STFR; Giacino et al. (2002)	EEG resting state	C-ApEn indices significantly lower in VS (*p* < 0.001)

*Abbreviation legend: VS = vegetative state; MCS = minimally conscious state; DTI = diffusion tension imaging; CRS-R = Coma Recovery Scale – Revised; RCP = Royal College of Physicians; M-STFR = Multi-Society Task Force Report; GCS = Glasgow Coma Scale; EEG = electroencephalography; PET = positron emission tomography; BIS = bispectral index; C-ApEn = cross-approximate entropy; n.s. = not significant*.

**Table 2:** **Stimulus-dependent behavioral assessments**.

Study	Number of subjects		Diagnostic criteria	Type of assessment	Stimuli	Difference between VS and MCS
	VS	MCS				
Trojano et al. (2012)	9	9	CRS-R	Visual pursuit	Moving target	n.s difference in duration of fixations; Lower proportion of fixations in VS patients (*p* < 0.001).

*Abbreviation legend: VS = vegetative state; MCS = minimally conscious state; CRS-R = Coma Recovery Scale – Revised; n.s. = not significant*.

**Table 3:** **Stimulus-dependent neurophysiological assessments: processing of sensory stimuli**.

Study	Number of subjects		Diagnostic criteria	Type of assessment	Stimuli	Difference between VS and MCS
	VS	MCS			
Boly et al. (2004)	15	5	M-STFR (1994); Giacino et al. (2002)	PET	Auditory (clicks)	n.s difference in BAEPs; Lower functional connectivity in VS (*p* <0.05)
Boly et al. (2008)	5	15	M-STFR (1994); Giacino et al. (2002)	PET	Somatosensory (electrical nociceptive)	Greater activation in “pain matrix” areas for MCS (*p* < 0.05)
Cavinato et al. (2011)	11	6	DRS	EEG	Auditory (oddball tones)	n.s.
de Biase et al. (2014)	27	5	CRS-R	EEG	Visual (flash)	n.s.
de Biase et al. (2014)	27	5	CRS-R	EEG	Somatosensory (electrical)	n.s.
de Biase et al. (2014)	27	5	CRS-R	EEG	Auditory	n.s.
Fischer et al. (2010)	16	11	CRS-R	EEG	Somatosensory (electrical)	n.s.
Fischer et al. (2010)	16	11	CRS-R	EEG	Auditory (oddball tones)	n.s difference in BAEPs; Pa component significantly more absent or reduced in VS (*p* <0.05)
King et al. (2013)	75	68	CRS-R	EEG	Auditory (tones)	Higher mutual information in MCS (*p* <0.0001)
Kotchoubey et al. (2005)	50	48	DRS	EEG	Auditory (simple tones)	N1 and P2 more frequent in MCS (*p* <0.05)
Kotchoubey et al. (2005)	50	48	DRS	EEG	Auditory (simple tones oddball)	N1 and P2 more frequent in MCS (*p* <0.05)
Kotchoubey et al. (2005)	50	48	DRS	EEG	Auditory (complex tones oddball)	N1 and P2 more frequent in MCS (*p* <0.05)
Kotchoubey et al. (2005)	50	48	DRS	EEG	Auditory (natural sounds)	N1 and P2 more frequent in MCS (*p* <0.05)
Kotchoubey et al. (2005)	50	48	DRS	EEG	Auditory (complex tones, MMN paradigm)	n.s.
Kotchoubey et al. (2005)	50	48	DRS	EEG	Auditory (simple tones, MMN paradigm)	n.s.
Ragazzoni et al. (2013)	8	5	M-STFR (1994); Giacino et al. (2002)	EEG	Somatosensory (electrical)	n.s.
Ragazzoni et al. (2013)	8	5	M-STFR (1994); Giacino et al. (2002)	EEG	TMS	Absence of contralateral TEPs more frequent in VS patients (*p* <0.015)
Wu et al. (2011b)	21	16	M-STFR (1994); Giacino et al. (2002)	EEG	Somatosensory (electrical nociceptive)	n.s.
Wu et al. (2011a)	20	30	M-STFR (1994); Giacino et al. (2002)	EEG	Somatosensory (electrical nociceptive)	C-ApEn indices significantly lower in VS (*p* <0.001)

*Abbreviation legend: VS = vegetative state; MCS = minimally conscious state; n.s. = not significant; CRS-R = Coma Recovery Scale – Revised; M-STFR = Multi-Society Task Force Report; DRS = Disability Rating Scale; EEG = electroencephalography; PET = positron emission tomography; TMS = transcranial magnetic stimulation; BAEPs = brainstem auditory evoked potentials; C-ApEn = cross-approximate entropy*.

**Table 4:** **Stimulus-dependent neurophysiological assessments: processing of meaningful stimuli**.

Study	Number of subjects		Diagnostic criteria	Type of assessment	Stimuli	Difference between VS and MCS
	VS	MCS				
Cavinato et al. (2011)	11	6	DRS	EEG	SON (oddball with tones)	n.s.
Cavinato et al. (2011)	11	6	DRS	EEG	SON (oddball with other names)	n.s.
Fellinger et al. (2011)	13	8	CRS-R	EEG	SON (oddball with other names), passive listening	n.s.
Fellinger et al. (2011)	13	8	CRS-R	EEG	SON (oddball with other names), attention to SON	n.s.
Fellinger et al. (2011)	13	8	CRS-R	EEG	SON (oddball with other names), attention to another name	n.s.
Fischer et al. (2010)	16	11	CRS-R	EEG	SON (oddball with tones)	n.s.
Kotchoubey et al. (2005)	50	48	DRS	EEG	Semantic (word categories)	n.s.
Kotchoubey et al. (2005)	50	48	DRS	EEG	Semantic (word pairs)	n.s.
Kotchoubey et al. (2005)	50	48	DRS	EEG	Semantic (sentences)	n.s.
Kotchoubey et al. (2013)	6	6	CRS-R	fMRI	Pain cries	Higher weighted global connectivity in MCS (*p* <0.05)
Kotchoubey et al. (2014)	29	26	CRS-R	fMRI	Semantic (sentences)	n.s.
Perrin et al. (2006)	5	6	CRS-R	EEG	SON	n.s.
Wu et al. (2011b)	21	16	M-STFR (1994); Giacino et al. (2002)	EEG	Music	n.s.
Wu et al. (2011a)	20	30	M-STFR (1994); Giacino et al. (2002)	EEG	Music	C-ApEn indices significantly lower in VS (*p* <0.001)
Wu et al. (2011a)	20	30	M-STFR (1994); Giacino et al. (2002)	EEG	Semantic (words)	C-ApEn indices significantly lower in VS (*p* <0.001)

*Abbreviation legend: VS = vegetative state; MCS = minimally conscious state; DRS = Disability Rating Scale; CRS-R = Coma Recovery Scale – Revised; M-STFR = Multi-Society Task Force Report; EEG = electroencephalography; fMRI = functional magnetic resonance; SON = subject’s own name; C-ApEn = cross-approximate entropy; n.s. = not significant*.

## Stimulus-independent physiological and neurophysiological assessments

### Resting state measurements

In order to overcome possible negative results due to assessments that imply the patients’ speech compliance and comprehension, considerable effort has been taken in the last years to find diagnostic indicators that are independent of task demands (Lechinger et al., 2013). Resting-state electroencephalography (EEG) qualifies as a relatively easy method that can be applied at bedside, and a high level of EEG complexity (i.e., variability) seems to be a good predictor of functional outcome (Fingelkurts et al., 2011).

Coleman et al. (2005) used simultaneous EEG and positron emission tomography (PET) to assess the integrity of the homeostatic coupling relationship between neuronal electrical function and cerebral metabolism, in patients with a VS (*n* = 6) and a MCS (*n* = 4) diagnosis. The coupling between neuronal electrical activity and regional glucose metabolism appeared to be preserved in all patients in MCS, but was absent in patients in VS. Whereas the VS group showed a higher degree of EEG slow wave activity compared to the MCS group, the regional cerebral metabolic rate, however, did not vary significantly between the two patient groups.

Lehembre et al. (2012) analyzed the differences in power spectra and connectivity in the resting state EEG of patients with a VS (*n* = 10) and a MCS (*n* = 18) diagnosis. In the delta band, relative power spectra were higher in the VS group compared to the MCS group. Conversely, in the alpha band, relative power spectra were higher in the MCS group compared to the VS group. These findings suggest a slowing of brain electrical activity in the patients with a VS diagnosis. Connectivity assessments based on classical coherence did not differentiate between the two diagnostic groups for any of the frequency bands. To diminish the influence of common sources due to volume conduction, the phase lag index (PLI) and the imaginary part of coherence (IC) were also computed (for more in-depth explanations of these indices, refer to the works of Nolte et al., 2004). The PLI and IC indicated a higher frontal-to-posterior connectivity in the theta band in the MCS group compared to the VS group. Importantly, as observed by the authors, these results were only obtained at the group level, so that further work would be required in the direction of disentangling VS and MCS at the individual level.

Differently from Coleman et al. (2005) and from Lehembre et al. (2012), Lechinger et al. (2013) did not find significant differences for any of the frequency bands in the EEG resting state of patients with a VS (*n* = 8) and a MCS (*n* = 9) diagnosis. Nevertheless, frequency ratios, and especially the alpha/theta ratio, appeared to be good predictors for CRS-R scores, as slower brain activity was associated to lower function. From this study, it could be concluded that although EEG frequency ratios may not be able to univocally discriminate between VS and MCS, they could indeed represent a good predictor of residual cognitive functioning.

In recent years, Bonfiglio et al. (2009) observed a bioelectrical oscillatory activity in the delta frequency range, which was both time-locked and phase-locked to the spontaneous blinking at rest. This activation, defined as blink-related delta oscillations (delta BROs), has been associated to a mechanism comparing the current environment image, appearing at the eyelid opening, with its mnestic representation at the moment of eyelid closure. Bonfiglio et al. (2013) hypothesized a relationship between the conscious state of a subject and his/her blinking behavior, interpreted as a signature of the automatic monitoring of the surrounding environment. Hence, they assessed whether BROs could allow discriminating between patients in VS (*n* = 4) and in MCS (*n* = 5) during resting state. In fact, delta BROs were absent or significantly reduced in both groups of patients compared to healthy individuals, and could not reliably differentiate between VS and MCS. Interestingly, a direct relationship between quantitative aspects of delta BROs and the Levels of Cognitive Functioning Scale (LCFS) was found. This result is consistent with the findings of Lechinger et al. (2013), showing that although resting state activation may not reliably discriminate between diagnostic categories, it indeed correlates with levels of cognitive functioning.

In a follow-up study on the same patients, Bonfiglio et al. (2014) found that alpha and beta bands were characterized by higher power in the MCS group compared to the VS group. For the interpretation of this finding, it is important to consider that three of the five subjects with a MCS diagnosis actually presented a higher LCFS score (<4) than what is considered typical for this diagnostic category, therefore showing relatively high cognitive functioning (MCS+).

In search of an objective measurement tool for achieving a more accurate assessment of patients in disorders of consciousness, Schnakers et al. (2008) computed the bispectral index (BIS) in patients with a VS (*n* = 13) and a MCS (*n* = 30) diagnosis who underwent an EEG resting-state measurement. The BIS, originally designed to measure the depth of anesthesia and sedation, is empirically derived from three resting state EEG parameters, namely (1) the ratio of the power in the beta ranges; (2) the ratio of the bicoherence in fast and slower frequencies provided by bispectral analysis; and (3) the burst suppression ratio. This index, normalized on a scale from 0 (no arousal) to 100 (patient fully aroused), was significantly lower in patients in VS (63/100) compared to patients in MCS (80/100). This result was in line with a positive correlation between the BIS and the consciousness scores obtained with the CRS-R.

During the past years, nonlinear dynamic analyses (NDA) have become a common way to study neural mechanisms, allowing the investigation of informative correlations such as the degree of synchronization within local networks, and the coupling between distant cortical networks (Wu et al., 2011a). Wu et al. (2011a) applied NDA on EEG resting state data collected in patients with VS (*n* = 21) and MCS (*n* = 16) diagnosis, using three nonlinear indices: (i) Lempel-Ziv complexity (LZC), which quantifies the complexity of time series, and has also been used for characterizing the depth of anesthesia (Zhang et al., 2001); (ii) Approximate entropy (ApEn), which quantifies the irregularity of data time series and is usually used for assessing whether the subject is awake (e.g., high levels of entropy during anesthesia demonstrate that the subject is awake, whereas low levels of entropy indicate unconsciousness); (iii) Cross-approximate entropy (C-ApEn), which measures the degree of dissimilarity between two concurrent series, allowing to uncover subtle disruptions in network dynamics. Although both patients in VS and in MCS showed significantly lower nonlinear indices compared to healthy controls, these indices did not allow differentiating VS and MCS. In a follow-up study on a larger sample of subjects (VS = 20, MCS = 30), the same group (Wu et al., 2011b) reported that the interconnection of local and distant cortical networks, measured with the C-ApEn index, was higher in the MCS group compared to the VS group.

Recently, diffusion tensor imaging (DTI) has been used to assess white matter integrity in patients with disorders of consciousness (Voss et al., 2006; Perlbarg et al., 2009; Tollard et al., 2009). Fernández-Espejo et al. (2011) used this technique to compare patients with a VS (*n* = 10) and MCS (*n* = 15) diagnosis. Significant abnormalities were observed in the integrity of the tissue in subcortical, thalamic and brainstem regions in both groups of patients. Compared to the MCS group, the VS group showed a significant decrease in the mean diffusivity (MD) of the subcortical white matter and of the thalamus, but not of the MD of the brainstem.

In sum, whereas some resting state assessments seem to be indeed able to differentiate between the VS and the MCS conditions, others evidently fail to do so. Nevertheless, a correlation between brain activity and behavioral assessments (on which the VS and MCS diagnoses are based) is generally present. This could indicate that although evident behavior can be a good predictor of brain activation, it might not always allow differentiating patients in two clear-cut categories.

### Assessment of sleep

The assessment of sleep could represent a useful stimulus-independent measure of a person’s level of consciousness. The presence of normal sleep in patients with a VS diagnosis, however, is still a matter of debate. For instance, Landsness et al. (2011) observed that patients with a VS diagnosis did not show typical sleep-related EEG patterns. Nevertheless, according to the Multi-Society Task Force for Permanent Vegetative State (PVS, 1994) and the Royal College of Physicians (Royal College of Physicians, 2003), wakefulness, i.e., the presence of typically cycling periods of eye-closure and eye-opening resembling sleep cycles, is preserved in both patients in VS and in MCS.

Cruse et al. (2013) assessed circadian sleep-wake rhythms in patients in VS (*n* = 18) and MCS (*n* = 37) during four days, using a wrist-mounted actigraphy device to measure the frequency and amplitude of motor activity. No significant effect of diagnosis was found on the proportion of patients exhibiting circadian rhythms, which were present in 83% of patients in VS and 84% of patients in MCS. Circadian rhythms, however, appeared to be weaker in VS relative to MCS, and motor activity positively correlated with the behavioral scores obtained with the CRS-R. What is particularly interesting in this study is that a significant proportion of patients, either with a VS or an MCS diagnosis, did not exhibit statistically reliable circadian sleep-wake rhythms, which, by definition, should be preserved in these diagnostic categories.

de Biase et al. (2014) assessed EEG sleep patterns during 24 h on patients diagnosed as in VS (*n* = 27) and as in MCS (*n* = 5). More specifically, the authors investigated the presence of a sleep-wake cycle, sleep spindles, rapid eye movement (REM), and K-complexes (i.e., indicators of non-REM sleep). All sleep elements were present at least once during the measurement in all patients in MCS, but only in 7.4% of patients in VS. This difference was significant, especially concerning the presence of REM sleep (100% in MCS and 14.8% in VS). In general, subjects showing all sleep elements had significantly higher CRS-R scores compared to those lacking some elements.

Although only two studies included a statistical comparison concerning sleep in patients with VS and MCS diagnoses, it appears that the differences between the two groups are not always marked, as in the study by Cruse et al. (2013). Nevertheless, these studies have in common a correlation between behavioral scores and sleep elements. Hence, although more studies comparing sleep in the two patient groups are required, it may be concluded that ordinal behavioral scores, rather than categories, could be more useful in predicting sleep elements.

## Stimulus-dependent behavioral assessments: visual pursuit

All guidelines for the diagnoses of disorders of consciousness consider visual tracking as an important marker for differentiating VS and MCS (Giacino et al., 2002; Majerus et al., 2005; Vanhaudenhuyse et al., 2008; Schnakers et al., 2009; Trojano et al., 2012). Whereas fixation of a stationary visual target is considered compatible with the diagnosis of VS (PVS, 1994; Royal College of Physicians, 2003; Bruno et al., 2010), the visual pursuit of moving stimuli is considered sufficient for the diagnosis of MCS (Giacino et al., 2002, 2004; Majerus et al., 2005; Schnakers et al., 2009). Assuming whether a patient’s behavior is volitional or reflexive, however, strongly depends on the examiner and on the different assessment tools. In order to obtain a more objective measure, Trojano et al. (2012) used an eye-tracker to quantify visual responses to moving stimuli in patients diagnosed as in VS (*n* = 9) and in MCS (*n* = 9). Participants were asked to keep their eyes on a moving target as much as possible throughout trials. The mean duration of single fixations, considered to be related to information processing, did not differ in the two diagnostic categories. The proportion of on- or off-target fixations was higher in patients in MCS, compared to patients in VS. It is nevertheless important to point out that the proportion of fixations was notably low in both groups of patients. Interestingly, two patients with a MCS diagnosis showed a fixation distribution that did not differ significantly from chance level, therefore conflicting with their diagnosis.

To our knowledge, this is the only behavioral assessment presenting a direct statistical comparison between the performance of patients with a VS and a MCS diagnosis. Albeit some difference between the two groups could be found, the very low proportion of fixations for the MCS group questions the validity of their diagnosis.

## Stimulus-dependent neurophysiological assessments: processing of sensory stimuli

In the last years, electrophysiological and neuroimaging assessments have been developed to accompany the behavioral evaluation of disorder of consciousness. Many of these measurements are based on the “hierarchic complexity hypothesis” (Kotchoubey, 2002; Kotchoubey et al., 2005), according to which the ability to process physically simple stimulus qualities (e.g., sounds) is a prerequisite for the processing of more complex stimuli (e.g., word meanings). Hence, according to this hypothesis, the presence of middle latency event-related potential (ERP) components (e.g., the N1 and the mismatch negativity (MMN)), which are related to the activity of the sensory cortex, should be a prerequisite for the presence of later components (e.g., the P300), reflecting the activity of associate cortical areas, which are therefore related to more complex processes. The validation of this hypothesis is particularly important, as its rationale is generally used in the diagnosis of disorders of consciousness. In fact, if a patient does not show early responses, the assessment is usually interrupted, such that the investigation of later responses related to more complex processing is not investigated. In the last years, several neurophysiological studies using different kinds of stimuli have been performed, and provide us with useful information about the hierarchic hypothesis.

### Auditory and visual stimulation

Boly’s research group (Boly et al., 2004) used O-radiolabeled PET to investigate the differences in cortico-cortical functional connectivity between patients with a VS (*n* = 15) and a MCS (*n* = 5) diagnosis, while stimulated with simple auditory clicks. The groups did not differ relatively to brainstem auditory evoked potentials (BAEP). In the MCS group, auditory stimulation activated the auditory cortex bilaterally, mostly in the transverse and superior temporal gyri (TTG, STG). The VS group presented a very similar activation pattern in the bilateral auditory cortex, although less widespread. Functional connectivity between the secondary auditory cortex and posterior temporal and prefrontal areas, considered to be involved in higher levels of auditory processing, was significantly tighter in patients in MCS compared to patients in VS. Boly et al. (2004) interpreted this finding as an evidence of the differences in attentional state and conscious perception between the two groups.

Fischer et al. (2010) used an auditory oddball paradigm in patients diagnosed as in VS (*n* = 16) and in MCS (*n* = 11). Similarly as in the study by Boly et al. (2004), BAEPs did not differ between the two groups. The Pa component of middle latency auditory evoked potentials (MLAEPs), however, was significantly more absent or reduced in the VS group compared to the MCS group, and N1 abolition was significantly more common in the VS group. The MMN did not differ between the two groups. Consistently with Boly et al. (2004) and with Fischer et al. (2010), de Biase et al. (2014) also did not find significant differences in the BAEPs of patients with VS (*n* = 27) and MCS (*n* = 5) diagnosis.

Kotchoubey et al. (2005) investigated how frequently ERP responses to auditory stimuli could be found in a large sample of patients in VS (*n* = 50) and in MCS (*n* = 48), with several different oddball and MMN paradigms. The EEG responses to the different kinds of stimuli were estimated as a function of complexity level of cortical processing. More precisely, the aim was to verify the hierarchic complexity hypothesis, according to which the processing of complex stimuli is always subtended to the processing of more simple stimuli. Participants were divided into two main groups (MG1, MG2) and two control groups (CG1, CG2). MG1 (*n* = 38) comprised patients with a VS diagnosis, with dominant theta or slow alpha EEG activity, not suppressed by light. MG2 (*n* = 38) comprised patients with a MCS diagnosis with a dominant theta background activity. CG1 (*n* = 12) comprised patients clinically and neuropsychologically identical those in MG1, but characterized by a more pathological rest EEG, with either large diffuse delta waves, a flat EEG, or alternation of delta activity and paroxysmal discharges. CG2 (*n* = 10) comprised patients who were similar to those in MG2, but whose EEG was characterized by either fast theta or slow alpha oscillations, which were suppressed by light. Three oddball paradigms were used, i.e., one with simple sine tones, one with complex tones (harmonic chords), and one with natural sounds (vowels). Moreover, two MMN paradigms were used, one with simple tones and the other with complex tones. These five paradigms were designed to assess three levels of cortical processes, namely auditory cortical responses (P1, N1, and P2), the MMN (as a sign of primary differentiation outside the focus of attention), and the P3 (indicating a deeper level of differentiation). No significant difference was found between the two main groups for the P1, N1, P2, and P3 components. MG1 and MG2 only differed significantly in the frequency of MMN, as unexpectedly, the MG1 characterized by a more severe diagnosis (i.e., VS), exhibited better MMN results. The differences with the respective control groups were more substantial, as both N1 and MMN responses were less frequent in CG1 than in MG1. CG2 presented more frequent P3 responses compared to MG2. Importantly, when compared regardless of their background activity, patients in VS and in MCS only differed significantly relative to early cortical responses (N1 and P2), which were more frequent in the MCS group.

The study of Kotchoubey et al. (2005) is particularly interesting, as the inspection of individual data showed that the hierarchical hypothesis is not universally confirmed. In fact, the N1-P2 complex did not reach significance in some patients who indeed presented a significant P3. Hence, the assertion that the processing of simple stimuli would be easier than that of physically complex stimuli was rebutted. Moreover, more complex responses were present above chance level not only in patients with a MCS diagnosis, but also in the ones with a VS diagnosis. The P3 and P600 components were found in a greater than chance percentage of patients in VS, suggesting activity in association cortical areas. These unexpected responses could be related to a modular structure of cortical information processing, in which encapsulated modules are suggested to exist and work, while disconnected from other modules, therefore leading to “islands’ of awareness in severely damaged patients.

The results obtained by Kotchoubey et al. (2005) are in disagreement with those of Boly et al. (2004), who reported tighter functional connectivity in patients in MCS, suggesting higher levels of auditory processing in these patients. Nevertheless, it should be observed that Boly et al. (2004) did not divide their samples into subgroups according to their background EEG activity. In the study by Kotchoubey et al. (2005), across all patients in VS, ERPs were slightly less than those for patients in MCS but these differences disappeared when patients with a similar EEG pattern were compared, indicating that background EEG should be controlled for when assessing functions in disorders of consciousness.

Cavinato et al. (2011) performed an oddball paradigm with sine tones on patients with a VS (*n* = 11) and a MCS (*n* = 6) diagnosis. Participants were instructed to count the deviant tones. No significant differences were found between groups concerning ERP amplitude and latency. Importantly, more than half of the patients in VS presented a reliable P3 component, indicating a spared modular cognitive ability to discriminate between simple sounds, or some fluctuation of awareness, despite the complete lack of externally observable behaviors. The authors suggested that the higher chance, compared to previous studies, of recording long-latency responses in patients in VS could have been affected by the use of salient stimuli, as well as by an active condition in which subjects were explicitly asked to keep a mental count of the number of target stimuli.

King et al. (2013) assessed global information sharing across brain areas in patients VS (*n* = 75) and in MCS (*n* = 68), who performed an EEG auditory paradigm for clinical purposes (listening to four identical tones). The authors introduced an index, the “weighted symbolic mutual information” (wSMI), namely the estimate of the amount of information shared by two EEG signals. The VS group presented a significantly lower wSMI than the MCS group. Such difference appeared to be particularly prominent across centroposterior areas, compatibly with theories associating consciousness with recurrent loops in posterior networks (Lamme, 2010).

The only comparison relative to visual evoked potentials (VEPs) was performed by de Biase et al. (2014), who used flash stimuli with patients with a VS (*n* = 27) and a MCS (*n* = 5) diagnosis. VEPs were not significantly different in the two groups.

These findings indicate that a differentiation between the VS and MCS categories on the basis of the processing of auditory and visual stimuli is not always possible (Kotchoubey et al., 2005; Cavinato et al., 2011; de Biase et al., 2014). Moreover, clear-cut differentiations between the two groups are complicated by the violation of the hierarchic hypothesis (Kotchoubey et al., 2005), such that patients with a VS diagnosis who do not present early EEG responses may indeed exhibit higher-latency responses relative to more complex processing, possibly due to spared modular cognitive abilities. Interestingly, asking participants to perform an active task (Cavinato et al., 2011) instead of passively perceiving the stimuli may decrease the possibility of differentiating between the two categories.

### Somatosensory, nociceptive, and transcranial magnetic stimulation

Boly et al. (2008) investigated nociception in patients in VS (*n* = 5) and in MCS (*n* = 15) following electrical stimulation, using PET. In the MCS group, the thalamus, the primary (S1) and secondary (S2) somatosensory cortices, the insula, the frontoparietal cortex, and the anterior cingulate cortex (ACC) were significantly more activated than in the VS group. These areas, belonging to the so-called “pain matrix”, have been recently associated to the ability to detect, orient attention towards, and react to the occurrence of salient sensory events (Iannetti and Mouraux, 2010; Legrain et al., 2011; Mouraux et al., 2011). Compared to the VS group, the MCS group showed a higher functional connectivity between the S1 and the lateral and medial frontotemporal areas.

Fischer et al. (2010) measured somatosensory evoked potentials (SEPs) in patients in VS (*n* = 16) and in MCS (*n* = 11), by electrically stimulating the median nerve. The abolition or the delay of the N20-P24 complex of SEPs was more frequent in the VS group than in the MCS group.

Ragazzoni et al. (2013) also measured SEPs elicited through electrical stimulation of the median nerve in patients with a VS (*n* = 8) and a MCS (*n* = 5) diagnosis. In contrast with Fischer et al. (2010), no significant difference emerged between the two patient groups concerning the N20 and N1 peaks. The EEG frequency power spectra also did not discriminate significantly between MCS and VS, in none of the frequency bands. With a similar experiment, de Biase et al. (2014) reported that SEPs in patients diagnosed as in VS (*n* = 27) and as in MCS (*n* = 5) during median nerve stimulation could not differentiate between the two groups.

Wu et al. (2011b) measured non-linear indices (LZC, ApEn, and C-ApEn, described previously in paragraph Stimulus-Independent Physiological and Neurophysiological Assessments) in the EEG of patients in VS (*n* = 21) and in MCS (*n* = 16) who underwent nociceptive acupuncture electrical stimulation. None of the indices differed significantly between the two patient groups. In a similar study performed by the same team on a different sample, however, the C-ApEn index was significantly lower in the VS group (Wu et al., 2011a).

With the same groups of subjects who underwent electrical stimulation, Ragazzoni et al. (2013) applied transcranial magnetic stimulation (TMS) to the scalp overlaying the primary motor cortex (M1) of the less affected hemisphere. Contralateral TMS-evoked potentials (TEPs) were significantly more absent in the VS compared to the MCS group, whereas no significant difference in the absence of ipsilateral TEPs emerged. As noted by the authors, TEPs and ERPs explore different aspects of brain function, i.e., TEPs directly assess basic properties of intra- and inter-hemispheric circuitries, whereas ERPs involve the activation of associative cortices and are specifically linked to cognitive processes.

Similarly to what emerged from resting state studies, sleep assessments, and investigations using auditory and visual stimulation, brain responses to somatosensory and nociceptive stimulation may allow discriminating between VS and MCS in some cases (Boly et al., 2008; Fischer et al., 2010; Wu et al., 2011b), but not in the totality of the assessments (Wu et al., 2011a; Ragazzoni et al., 2013; de Biase et al., 2014).

## Stimulus-dependent neurophysiological assessments: processing of meaningful stimuli

### Affective stimulation and music

Few studies have been performed using non-verbal stimuli, which may however be meaningful for the subjects, such as human vocalizations or music (Wu et al., 2011b; Kotchoubey et al., 2013). For instance, Kotchoubey et al. (2013) compared fMRI connectivity in patients with a VS (*n* = 6) and a MCS (*n* = 6) diagnosis, who were presented with an emotional empathy task, namely listening to emotional cries of pain and suffering. The stimuli were drawn from the International Affective Digitized Sounds (IADS) database, and had been rated by healthy individuals as highly emotional. No significant difference in task-related activation was found between the two patient groups. Global functional connectivity, however, was significantly higher in the MCS group compared to the VS group, particularly in clusters that have been associated to empathy, such as the insula, the ACC, and the cerebellum.

Wu et al. (2011b) compared non-linear dynamic EEG indices in patients in VS (*n* = 21) and in MCS (*n* = 16), who received musical stimulation, and reported no significant differences between the two groups in the LZC, ApEn, and C-ApEn indices. In another study from the same team (2011b), the C-ApEn index was able to discriminate between the two patient groups (VS = 20, MCS = 30), as it was significantly higher in the MCS group.

Given the limited number of studies assessing the processing of non-verbal meaningful stimuli, which appear to be effective in differentiating levels of consciousness, further investigations should be carried out in this direction.

### Semantic stimuli

Understanding spoken language is a complex ability. Firstly, listeners must analyze the acoustic properties of speech, to identify individual linguistic units. Secondly, they have to retrieve the stored representations of word meanings from memory. Finally, such representations should be adequately combined to build a representation of the whole sentence’s meaning (McClelland and Elman, 1986). Consistently with the hierarchical hypothesis, results from functional neuroimaging studies in healthy volunteers support the idea of processing streams radiating from primary auditory regions with low-level acoustic processes located in and around the auditory cortex, whereas higher level linguistic processes involved in computing meanings are located further away from primary auditory regions, in temporal and frontal regions (Belin et al., 2000; Binder et al., 2000; Scott et al., 2000; Davis and Johnsrude, 2003; Scott and Johnsrude, 2003; Rodd et al., 2005).

Kotchoubey et al. (2005) tested the hierarchic hypothesis relatively to language comprehension using three semantic oddball paradigms (with word categories, word pairs, and sentences), with the same sample of patients as in the auditory experiments described in paragraph Somatosensory, Nociceptive, and Transcranial Magnetic Stimulation (VS = 50, MCS = 48), divided into four groups according to their background EEG activity (MG1 = 38, MG2 = 38, CG1 = 12, CG2 = 10). Semantic responses were never found in patients in CG1, namely patients with a VS diagnosis and an abnormal EEG background activity. Concerning the main groups of patients in VS and in MCS, no significant difference was found in the percentage of patients showing N400 responses. Moreover, in contrast to the hierarchical hypothesis, some patients who had not shown a significant oddball P3 or a MMN, presented indeed a significant N400. In fact, cortical evidence for semantic differentiation was found in around 25% of the patients with a VS diagnosis. Importantly, Kotchoubey et al. (2005) pointed out that the processing capacities of these patients could have been underestimated, and that the ERP data might have been biased toward false negatives, mainly for two reasons: firstly, several factors such as habituation, fluctuations in arousal, and fatigue increase the probability of missing an ERP component (Neumann and Kotchoubey, 2004); secondly, an ERP component could be lacking due to a focal lesion to its cortical generator, irrespectively of the state of consciousness. The hierarchy violations in this study may be partly explained by possible fluctuations of patients’ arousal, such that some experiments may have been performed in more “favorable” periods than others. Another possible explanation is that the responses could be related to a modular structure of cortical information processing, in which individual encapsulated cortical modules work independently from other modules.

In a recent fMRI study, Kotchoubey et al. (2014) presented patients with a VS (*n* = 29) and a MCS (*n* = 26) diagnosis with short sentences, half of which were factually correct, and the other half factually incorrect. Differential activation for false and true sentences was found in 38% of the patients with a VS diagnosis, and for 19% of the patients with a MCS diagnosis, in language-related regions including Broca and Wernicke areas. Hence, in a considerable number of patients, responses closely resembled the ones of healthy individuals, albeit weaker. The difference between the two diagnostic groups, however, was not significant, consistently with the previous results obtained by the same research group (Kotchoubey et al., 2005).

Differently from Kotchoubey et al. (2005, 2014), who could not differentiate between semantic-related EEG responses in patients in VS and in MCS, Wu et al. (2011b) found that when presenting commonly used words to patients with a VS (*n* = 20) and a MCS diagnosis (*n* = 30), the C-ApEn non-linear index was significantly higher in the MCS group compared to the VS group.

Similarly to neurophysiological studies using simple sensory stimulation, experiments in which verbal stimuli are used also show that the VS and MCS categories are not always discriminated reliably, even when the considered samples are relatively large, and multiple assessments are performed (Kotchoubey et al., 2005, 2014). These experiments also represent further evidence of the fact that the hierarchic hypothesis can be violated. The computation of non-linear indices (Wu et al., 2011b), although still not widespread, may represent a useful strategy for assessing levels of consciousness, and could therefore become a useful integration of the assessment of ERPs.

#### Subject’s own name

A particular kind of semantic stimulus that is frequently used in the assessment of consciousness is the subject’s own name (SON). The SON is a word that is processed since infancy, and being self-referential, is considered to be more salient (Fellinger et al., 2011) and emotionally charged (Perrin et al., 2006) than other words. Moreover, compared to other stimuli, the SON has been reported to elicit enhanced cortical activity (Cavinato et al., 2011).

Perrin et al. (2006) investigated the detection of the SON, compared to other seven equiprobable first names (OFNs), in patients in VS (*n* = 5), and in patients in MCS (*n* = 6), as well as in patients in locked-in states (LIS, *n* = 4). A P3 component was observed in all patients in MCS and in LIS, and in three of the five patients in VS. The P3 amplitude was always significantly higher in response to one’s own name compared to other first names. However, this response did not allow differentiating VS from MCS. Interestingly, this was the first study that showed that a differential P3 component could be recorded in response to the SON, not only in patients with a diagnosis of MCS, but also in some patients diagnosed as in VS.

Fischer et al. (2010) performed an oddball paradigm in which the SON represented the deviant stimulus within a series of simple tones, with patients in VS (*n* = 16) and in MCS (*n* = 11). Consistently with Perrin et al. (2006), this task did not allow differentiating the two groups of patients, also because the novelty P3 component was absent in more than two thirds of the subjects, regardless their diagnosis. Similarly, Cavinato et al. (2011) used two SON oddball paradigms with patients diagnosed as in VS (*n* = 11) and in MCS (*n* = 6). In the first paradigm, the name of the participant was a deviant stimulus within a series of tone bursts. In the second paradigm, the SON was a deviant and three other first names were used as control stimuli. Participants were asked to count the number of times they heard their own name. No significant group effects for the ERP amplitude and latencies emerged from either experiment. Importantly, P3 responses were detected in more than half of the patients in VS. The high proportion of patients showing a response to saliency, despite the lack of observable behavior, could be explained by the fact that they were required to perform an active task.

Fellinger et al. (2011) used a selective attention paradigm in which three different conditions were used. In the first condition, patients in VS (*n* = 8) and in MCS (*n* = 13) had to passively listen to a list of first names, also comprising the SON. In the two active conditions, the participants were asked to either count the number of occurrences of the SON, or to count the occurrence of another specified name. In general, no significant differences were observed in the two groups of patients, as both groups exhibited a stronger theta-synchronization to their own names when forced to count them, and a delay in theta power in response to targets relative to non-targets when participants were instructed to count their own name. The only difference between the VS and the MCS groups was the time-point in which they reached the maximal increase in theta power for targets as compared to non-targets, since patients in VS exhibited a higher time jitter. In this study, both groups of patients showed to be capable of neuronally responding to their own name with an increase in theta activation, which was not present when they were asked to attend to other names or during passive listening. According to these results, and in agreement with Cavinato et al. (2011), both groups of patients were able to respond to the instruction of attending their own name, therefore using their working memory and performing some kind of top-down processing task.

These results represent a further confirmation of the fact that differentiating VS and MCS according to the patients’ brain responses is not at all straightforward, even when a salient, self-referential and emotionally-charged stimulus such as one’s own name is used as input.

## Discussion

Our literature review indicates that out of 47 measurements, 24 failed to reveal any significant statistical difference between behavioral or neurophysiological responses of patients with a VS and a MCS diagnosis. This is a considerable number, which might possibly be underestimated by a bias concerning the publication of negative results (Dickersin and Min, 1993; Jennings and Van Horn, 2012; Dwan et al., 2013; Kicinski, 2013). In fact, Dickersin (1990, 1997; Dickersin et al., 1992) estimated that statistically significant results are three times more likely to be published than null results. Although there is currently no available data on publication bias towards positive results specifically in the field of disorders of consciousness, such bias has been reported in adjacent medical fields, e.g., in the dissemination of clinical trial results (Dickersin and Min, 1993; van Lent et al., 2014). According to a recent survey performed by Malički and Marusić (2014), 36% of researchers reported unpublished trials due to a publication bias, and 30% admitted selective outcome reporting depending on their results. Moreover, research without statistically significant results takes longer time to gain publication than research with significant results (Stern and Simes, 1997; Ioannidis, 1998; Decullier et al., 2005; Scherer et al., 2007). An investigation on the publication of biomedical research protocols performed by Decullier et al. (2005) showed a highly significant difference (*p* < 0.001) in the time of publication from the date of committee approval, dependent on study outcomes (5.2 years for confirmatory results, 6.9 years for invalidating results, and 6.5 for inconclusive results). These results suggest that there could be more studies not showing statistical difference between VS and MCS, which have not been reported in the literature.

The studies described in this review involved different numbers of subjects, ranging from measurements comprising a total of nine patients, to studies with almost one hundred patients. Notwithstanding, no correlation emerged between the number of patients involved in the studies and the lack of significant differences between diagnostic groups (*r* = 0.12, *p* = 0.41).

The different experiments were performed on patients diagnosed in Italy (12 measurements), Germany (11 measurements), United Kingdom (3 measurements), Belgium (6 measurements), France (4 measurements), Austria (4 measurements), and China (7 measurements). Interestingly, the proportion of non-significant results markedly differed between countries (Italy: 67%, Germany: 55%, United Kingdom: 33%, Belgium: 17%, France: 50%, Austria: 100%, China: 43%), possibly indicating high variability in the application of standardized diagnostic criteria across countries. The interpretation of these percentages, however, is complicated not only by the difference in the number of studies, but mostly by the fact that multiple measurements were sometimes performed by the same research group, as in the case of the studies performed in Germany (Kotchoubey et al., 2005, 2013, 2014), often presenting non-significant differences between diagnostic groups, and in Belgium (Boly et al., 2004, 2008; Perrin et al., 2006; Schnakers et al., 2008; Lehembre et al., 2012; Cruse et al., 2013), where on the contrary, such differences generally emerged. Measurements in Italy, on the other hand, were performed by four independent research groups (Cavinato et al., 2011; Trojano et al., 2012; Bonfiglio et al., 2013, 2014; Ragazzoni et al., 2013; de Biase et al., 2014), and the occurrence of non-significant differences was common in all the groups, with only one exception (Trojano et al., 2012). A possible interpretation of these data could rely on the variability of the diagnoses across different hospitals and research groups. Nevertheless, it is not possible to exclude that the dissemination of negative results might be more encouraged in some laboratories compared to others.

Interestingly, a high percentage of non-significant differences between VS and MCS emerged from studies using meaningful stimuli, such as verbal stimuli and the SON, in which a markedly higher performance of patients with a MCS diagnosis was expected. It is important to note that, in several cases, patients’ behavioral or physiological responses contradicted their diagnosis, and therefore their assignment to either the VS or the MCS category. For instance, Cruse et al. (2013) did not find, in several patients, reliable circadian sleep-wake rhythms, which should be preserved in both VS and MCS by definition. Trojano et al. (2012) reported that fixation behavior, expected in both VS and MCS, was lacking in several patients. In some cases, patients with a VS diagnosis showed stronger electrophysiological responses than patients with a MCS diagnosis (i.e., for the MMN response), which also suggested the processing of complex stimuli characteristics (Kotchoubey et al., 2005). In particular, in the experiments performed by Kotchoubey et al. (2005), background EEG activity was more predictive of stimulus-related brain responses than clinical diagnosis. The results by Cavinato et al. (2011) indicate that not only patients with a MCS diagnosis, but also those with a VS diagnosis may be able to perform active tasks, therefore exhibiting purposeful behavior. P3 responses, generally considered to be absent in patients in VS, were nevertheless obtained in patients with a VS diagnosis in several assessments (Kotchoubey et al., 2005; Perrin et al., 2006).

A first interpretation of these findings could be that they derive from a high rate of misdiagnoses, so that many patients classified as in VS may actually be in a MCS condition, or vice versa. Misdiagnoses could be due to multiple reasons, such as the fluctuations of the patients’ arousal and conditions, fatigue, variations in medical and physical management, and inexperience of the evaluators (Neumann and Kotchoubey, 2004; Majerus et al., 2005; Gill-Thwaites, 2006; Schnakers et al., 2009; Gosseries et al., 2011). Nevertheless, the diagnoses of disorders of consciousness are generally performed on the basis of behavioral measures such as the CRS-R, which generally correlate with the presence of behavioral responses/electrophysiological activations (Schnakers et al., 2008; Bonfiglio et al., 2009, 2014; Lechinger et al., 2013; de Biase et al., 2014), even when these responses and activations are not predicted by clinical diagnosis (Bonfiglio et al., 2009; Lechinger et al., 2013). This observation inevitably questions the usefulness of using the VS and MCS dichotomic diagnostic categories, instead of relying on ordinal behavioral scores, which appear to have a stronger relationship with the patients’ responses. This is also in agreement with a recent multi-center observational study (Sattin et al., 2014), according to which patients in VS and in MCS often present more similarities than differences, and that interventions and long-term health care programs should be carried out mainly according to the descriptions of the patients’ functioning. It should also be considered that, in some cases, patients included in the MCS category have higher cognitive functioning scores compared to what would be considered normal for their diagnosis (Bonfiglio et al., 2014), although this might not be always indicated in research reports. In fact, some of the investigations which appear to discriminate between VS and MCS categories may be rather discriminating between groups with very different levels of cognitive functioning. Further complications derive from the use of different diagnostic criteria, also within the same study.

The violation of the hierarchic hypothesis in several measurements (Kotchoubey et al., 2005) indicates that the assessment of consciousness is not at all straightforward, and that individuals who appear to be unresponsive at the behavioral or at the electrophysiological level could indeed exhibit “islands” of awareness. These results are consistent with other reports showing that the processing of simple stimuli is not necessarily easier than that of more complex stimuli (Rauschecker, 1997; Tervaniemi et al., 2000; Hall et al., 2002), and that partially functional cerebral regions may be preserved even in brains with extremely severe injury (Schiff et al., 2002; Coleman et al., 2007). Hence, it is absolutely insufficient to declare a patient as “cortically non-responsive” when obtaining a negative result, as the same patient could exhibit significant responses in more demanding and complex tasks. These findings introduce the necessity of assessing patients on multiple aspects (i.e., behavioral responses, electrophysiological responses to simple sensory stimuli, electrophysiological responses to complex meaningful stimuli, etc.), which might lead to independent outcomes. Recently, Casali et al. (2013) introduced a promising non-invasive diagnostic approach based on the computation of the perturbational complexity index (PCI), which reflects the information content of the brain’s response to TMS (also found to be useful for the evaluation of levels of consciousness by Ragazzoni et al., 2013). The PCI appears to be correlated with the level of consciousness of healthy individuals during wakefulness, dreaming, non-REM sleep, and anesthesia, as well as of patients who emerged from coma, and could therefore be used as an integration of other neurophysiological measures.

The systematic use of multiple behavioral and neurophysiological assessment procedures could also be complemented by learning assessment strategies. Signs of learning, defined as a process by which a person acquires knowledge on the relationship between responses and environmental events (Bosco et al., 2009), might represent a basic level of non-reflective consciousness (Bosco et al., 2009, 2010). To this end, Bekinschtein et al. (2009) demonstrated that some patients diagnosed as in VS are able to learn associations between stimuli, and concluded that these individuals might have a partially preserved conscious processing, which cannot be detected by standard behavioral assessments. Lancioni et al. (2007, 2008a,b,c, 2009a,b) assessed learning in people with disorders of consciousness using a non-verbal procedure, technically supported by microswitches detecting simple responses (e.g., minimal forehead wrinkling, eye-blinking, slight movements, etc.) and electronic control systems providing the person with contingent feedback to these responses. These assessments also have the advantage of being less dependent on subjectivity compared to naked-eye examinations, while being less invasive than neurophysiological assessments. Moreover, learning procedures may also have the benefit of overcoming the extinction of goal-directed thinking in individuals with disorders of consciousness, allowing the performance of tasks that could facilitate the interaction of the patients with their environment (Liberati and Birbaumer, 2012).

The pertinence of placing a dividing line between VS and MCS was also recently debated by Nettleton et al. (2014), who introduced the concept of “diagnostic illusory”, which stresses the ambiguity and tensions deriving from the biomedical imperative to classify patients with disorders of consciousness in discrete and rigid categories. Diversified assessment procedures would allow developing a multifaceted evaluation, instead of a dichotomic categorization.

## Conclusions

From the analysis of several measures comparing VS and MCS, both at the behavioral and at the neurophysiological level, it emerges that there is no combination of variables which reliably and consistently allows discriminating between these two diagnostic categories. This pattern of results questions the empirical validity of the distinction between VS and MCS. We therefore suggest to overcome the use of a strict and clear-cut distinction between the two categories, and recommend the use of diversified assessment procedures (i.e., investigating behavioral responses, brain activation, learning process, etc.) in order to develop a multifaceted evaluation of patients with disorders of consciousness.

## Conflict of interest statement

The Associate Editor Giulio E. Lancioni declares that, despite having published in the past with author Marta Olivetti the review process was handled objectively and no conflict of interest exists. The authors declare that the research was conducted in the absence of any commercial or financial relationships that could be construed as a potential conflict of interest.
